# Differences in composition of interdigital skin microbiota predict sheep and feet that develop footrot

**DOI:** 10.1038/s41598-022-12772-7

**Published:** 2022-05-27

**Authors:** Rachel Clifton, Emma M. Monaghan, Martin J. Green, Kevin J. Purdy, Laura E. Green

**Affiliations:** 1 Institute of Microbiology and Infection, University of Birmingham, Edgbaston, UK; 2 School of Veterinary Medicine and Science, University of Nottingham, Sutton Bonington Campus, Leicestershire, UK; 3 School of Life Sciences, University of Warwick, Coventry, UK

**Keywords:** Metagenomics, Pathogenesis, Bacterial pathogenesis, Microbiome

## Abstract

Footrot has a major impact on health and productivity of sheep worldwide. The current paradigm for footrot pathogenesis is that physical damage to the interdigital skin (IDS) facilitates invasion of the essential pathogen *Dichelobacter nodosus*. The composition of the IDS microbiota is different in healthy and diseased feet, so an alternative hypothesis is that changes in the IDS microbiota facilitate footrot. We investigated the composition and diversity of the IDS microbiota of ten sheep, five that did develop footrot and five that did not (healthy) at weekly intervals for 20 weeks. The IDS microbiota was less diverse on sheep 2 + weeks before they developed footrot than on healthy sheep. This change could be explained by only seven of > 2000 bacterial taxa detected. The incubation period of footrot is 8–10 days, and there was a further reduction in microbial diversity on feet that developed footrot in that incubation period. We conclude that there are two stages of dysbiosis in footrot: the first predisposes sheep to footrot and the second occurs in feet during the incubation of footrot. These findings represent a step change in our understanding of the role of the IDS microbiota in footrot pathogenesis.

## Introduction

Ovine footrot is an infectious dermatitis of the interdigital skin (IDS) of sheep that causes lameness, reducing health and productivity in sheep worldwide^1–4^. In the UK, footrot is endemic^5^ with > 99% flocks affected^6^ and an average within-flock prevalence of footrot of 3.5%^7^. Approximately 3 million sheep (20% of the breeding population) become lame each year^8^ with economic losses of £24–£80 million per annum^3,9^.

Footrot is an inflammatory disease of the IDS with a complex pathogenesis. The Gram-negative anaerobe *Dichelobacter nodosus* has been identified as the essential pathogen and possesses virulence factors required for development of characteristic footrot lesions^10,11^. Importantly, *D. nodosus* is unable to invade the intact IDS and damage to the skin by some predisposing factor is required to initiate disease^10^. In early experimental models of footrot this was achieved by scarification of the skin^10,12^, or keeping sheep in pens heavily contaminated by faeces^13^, however, neither of these are realistic representations of the natural conditions in which disease occurs. More recently, McPherson et al.^14^ created a pasture-based model in which footrot could be induced by keeping sheep in wet conditions. Water maceration of feet induces hyperkeratosis in the hoof horn adjacent to the skin-horn junction, and this is believed to contribute to facilitating invasion by *D. nodosus*^15^. Under natural conditions footrot is more prevalent on wet pastures^10,16^, this is thought to be due to both the increased transmissibility of *D. nodosus* and increased susceptibility of feet to infection^15^.

In the 1960s it was proposed that the opportunistic pathogen *Fusobacterium necrophorum* facilitated invasion of the IDS by *D. nodosus*^12,13^, and whilst this was observed in experimental disease models^12^, there are several studies that report that in natural pathogenesis of disease *F. necrophorum* invades and proliferates only once footrot lesions have developed^10,12,17–19^. In addition, *F. necrophorum* is not present on all feet with footrot^18–20^ and is one of several organisms such as Spirochaetes^10^ and *Porphyromonas*^21,22^ that are found at increased abundance on diseased feet.

The gingival crevice is similar to IDS in that it is where ‘dead’ tooth and ‘dead’ horn abut living skin. Both sites are moist and have areas that are less oxygenated than others. The current paradigm for the pathogenesis of periodontitis is that the keystone pathogen *Porphyromonas gingivalis* facilitates the development of a dysbiotic microbial community; this then promotes an inflammatory response in the host^23–26^. It has been shown that a dysbiotic microbiota is present on feet affected by footrot^21,22^, and given the similarity between the IDS and the gingival crevice, one hypothesis is that dysbiosis of the IDS microbiota could cause inflammation of the IDS and therefore be an alternative predisposing factor for invasion by *D. nodosus*. However, it is currently unknown whether dysbiosis occurs prior to initiation of footrot, or secondary to the inflammation associated with footrot.

As well as advancing scientific knowledge, understanding whether the IDS microbiota has a causal role in pathogenesis of footrot could provide new approaches to managements for control of footrot. In the UK, farmers currently control footrot using a combination of antimicrobial treatment of diseased sheep^3,27^, biosecurity and a mildly effective vaccine^28^. Treatment of footrot accounts for 65% of antibiotic use on sheep farms in the UK ^29^ and controlling footrot requires constant management. Many UK farmers find the managements onerous^30^ and would prefer a more simple, efficient, alternative disease control tool^31^.

The aim of our study was to investigate the IDS microbiota before and during footrot to elucidate whether there is a role of the microbiota in disease initiation.

## Results

### Selection of sheep and definition of diseased and healthy feet and sheep

**Figure 1:**
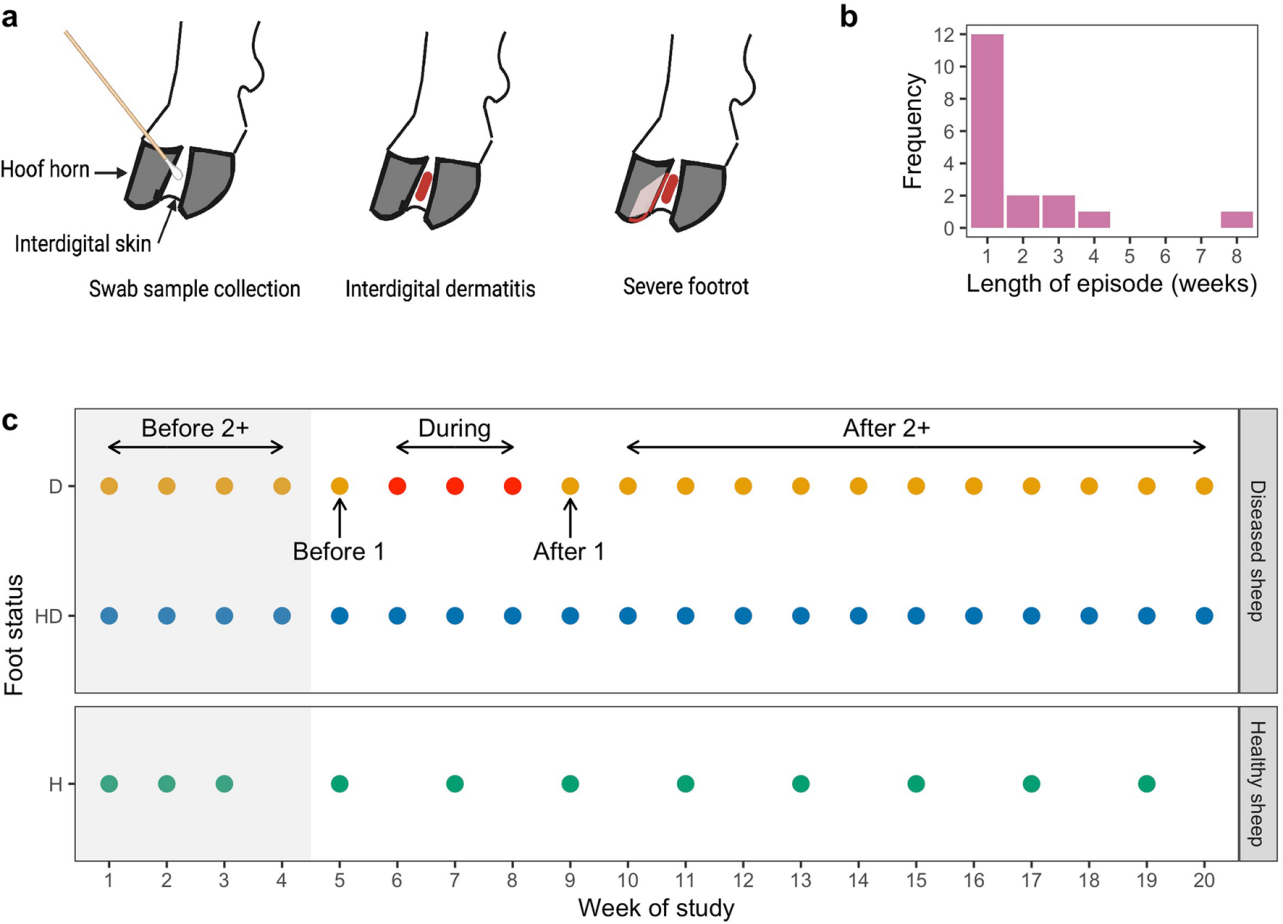
Classification of disease status of sheep and feet. (**a**) Illustration of location of swab sample collection, and distribution of lesions of interdigital dermatitis and severe footrot on the sheep foot. (**b**) Distribution of length of 18 footrot episodes in the 12 feet with footrot (D feet). (**c**) Schematic of classification of foot status. Coloured circles show time points at which samples were analysed for three classes of foot: D (orange) = diseased foot; HD (blue) = healthy foot of diseased sheep; H (green) = healthy foot of healthy sheep. An example of a footrot episode is shown in red, and temporal classification of samples from the D foot by time relative to this episode used in the analysis is shown by arrows above and below. Before 2 +  =  ≥ 2 weeks before footrot episode; before 1 = 1 week before footrot episode; during = during any footrot episode; after 1 = 1 week after footrot episode, after 2 +  =  ≥ 2 weeks after footrot episode. Grey shaded area indicates samples included in machine learning analysis for prediction of future disease status.

We observed the 160 feet of 40 sheep from one lowland footrot-affected flock in Warwickshire, UK, weekly for 20 weeks. Footrot phenotype^32^ and a swab sample from the IDS (Fig. 1a) were collected each week from each foot^19^. After the study was complete a subset of 10 sheep were selected for sequencing analysis of foot swabs (Fig. S1). This comprised five sheep with 20 healthy feet (H—healthy foot from healthy sheep) throughout the 20 week study and five sheep that developed footrot in 12 feet (D—diseased foot at some point in the 20 weeks of diseased sheep) with the other eight feet remaining healthy throughout the study (HD—healthy feet of diseased sheep) (Fig. 1c). For H feet, only samples from alternate weeks were analysed for the period from week 3–20 (Fig. 1c). Footrot was defined as inflammation of the IDS (interdigital dermatitis) score > 1^32^ and/or any separation of hoof horn from the living dermis (severe footrot) (Fig. 1a). There were 18 episodes of footrot in the 12 diseased feet that lasted 1–8 weeks (Fig. 1b), with a new episode being defined as an occurrence of footrot following two consecutive healthy scores. These episodes of footrot occurred from week 3 onwards (Fig. S1).

### The microbiota of the interdigital skin is temporally dynamic

There were 620 swab samples; 400 samples from sheep with footrot (D and HD feet) from each of the 20 weeks, and 220 samples from sheep that remained healthy (H) from weeks 1–3 and then 8 alternate weeks (Fig. 1c); 16S rRNA gene sequencing data from 603 samples (218-H, 229-D and 156-HD) were analysed. A total of 2723 bacterial operational taxonomic units (OTUs) were identified across all 603 samples. For 409 samples (91-H, 182-D and 136-HD), additional data on bacterial load of *D. nodosus* estimated by qPCR^18^ from a previous study were used^19^.

**Figure 2:**
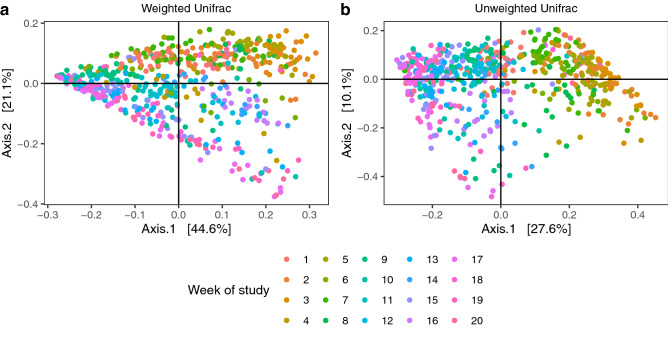
Temporal changes in interdigital skin microbial community composition. Principal coordinate analysis (PCoA) plot of (**a**) weighted and (**b**) unweighted Unifrac dissimilarities between microbial communities at the operational taxonomic unit (OTU) level. Each dot represents a sample coloured by week of study. Number of samples (n) = 603.

Initially we examined the level of similarity across all microbial communities of H, HD and D feet using four different distance measures: root Jensen Shannon distance (rJSD), Bray Curtis, and weighted and unweighted Unifrac. The IDS microbiota changed over time with all measures (Figs. 2 and S2), with communities that were temporally close more similar to each other than those that were temporally distant. Patterns were consistent across all feet irrespective of sheep or feet definition, indicating an external influence, possibly the environment, since changes correlated with change in soil moisture (Fig. S3). There was no evidence of samples clustering by foot status or sheep (Figs. S4 and S5).

### Composition of healthy feet IDS microbiota is predictive of which sheep subsequently develop footrot

To determine whether composition of the IDS microbiota was predictive of which sheep or feet subsequently developed footrot, we used a supervised machine learning approach. Samples from feet for sheep that developed footrot (D and HD) 2 + weeks before the first episode of disease occurred in that sheep were selected (week 1 for sheep 3535, weeks 1–2 for sheep 3514 and weeks 1–4 for sheep 3478, 3488 and 3547). Samples from H feet from all five healthy sheep from the same time period were also included (weeks 1–3; Fig. 1c) providing a total of 117 samples (60-H, 32-D and 25-HD) for classification. Because time was linked to OTUs (Fig. 2), a random forest model was used to identify OTUs that were strongly correlated with time in H feet and those OTUs were removed from the dataset prior to analysis.

**Figure 3:**
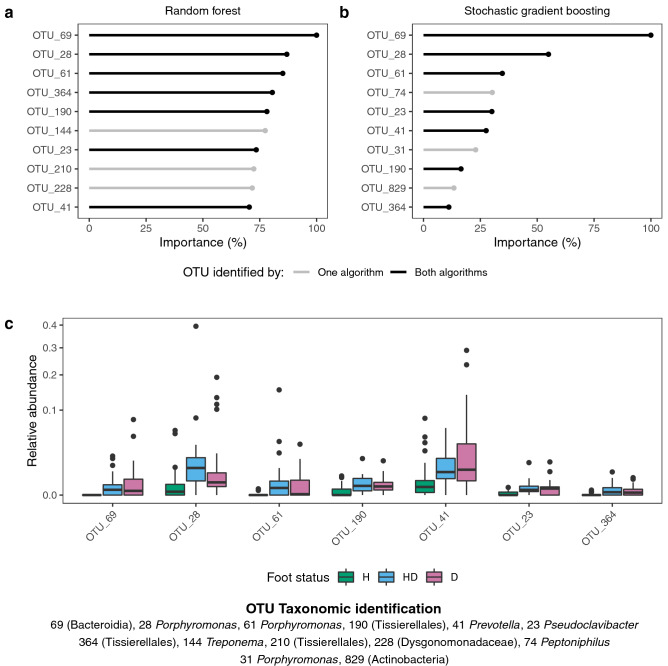
Composition of the IDS microbiota is predictive of footrot status of sheep. Importance of features retained in (**a**) random forest and (**b**) stochastic gradient boosting model for predicting disease status. Importance is scaled as a percentage relative to the importance of the most important feature. (**c**) Boxplots of relative abundance by foot status for operational taxonomic units (OTUs) identified by both random forest and stochastic gradient boosting model. H = healthy foot of healthy sheep; HD = healthy foot of diseased sheep; D = diseased foot. Number of samples (n) = 117.

We initially implemented random forest and stochastic gradient boosting algorithms with recursive feature elimination to identify OTUs that classified H feet as belonging to healthy sheep and HD and D feet as belonging to diseased sheep. To increase robustness^33^, only OTUs identified by both algorithms were considered important. Sheep that developed footrot were differentiated from sheep that did not with a mean cross-validation accuracy of 0.71 for the random forest algorithm and 0.69 for the stochastic gradient boosting algorithm. Seven of the 2723 OTUs were identified by both algorithms (Fig. 3a and b); all seven were more present and abundant on D and HD than H feet (Table 1 and Fig. 3c).

**Table 1:** Frequency of detection of seven OTUs used to predict disease status on D, HD and H feet in the period 2 + weeks before footrot occurred.

	Taxonomic identification	Percentage of samples with OTU detected		
		D feet (n = 32)	HD feet (n = 25)	H feet (n = 60)
OTU_41	*Prevotella*	84	92	77
OTU_190	(Tissierellales)	84	96	40
OTU_69	(Bacteroidia)	53	56	0
OTU_28	*Porphyromonas*	94	96	58
OTU_23	*Pseudoclavibacter*	72	76	35
OTU_61	*Porphyromonas*	50	60	3
OTU_364	(Tissierellales)	59	56	7

**Figure 4:**
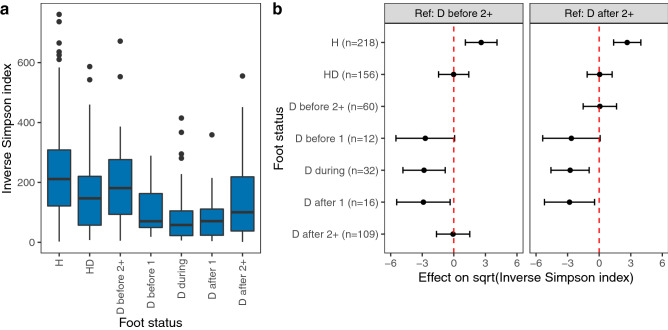
Diversity of the interdigital skin microbiota is temporally associated with footrot. (**a**) Boxplots of Inverse Simpson index in samples from all sheep by foot status. (**b**) Effects of foot status on square root transformed Inverse Simpson index as quantified by linear mixed-effects models. Centres indicate estimated fixed effects with error bars showing 95% confidence intervals. The foot status category used as the reference category in each model is shown above each plot. H = healthy foot of healthy sheep; HD = healthy foot of diseased sheep; D = diseased foot: before 2 +  =  ≥ 2 weeks before footrot episode; before 1 = 1 week before footrot episode; during = during any footrot episode in that foot; after 1 = 1 week after footrot episode, after 2 +  =  ≥ 2 weeks after footrot episode. Number of samples (n) = 603.

We then compared the performance of our original two-class model with a three-class random forest model to classify samples as belonging to H, HD or D feet. Mean cross-validation accuracy of the three-class model was 0.58 and whilst it correctly identified 57/60 samples from H feet, it performed poorly in identifying samples from HD and D feet (5/25 and 12/32 identified correctly, respectively). In addition, nine of the ten most important features from the three-class model were amongst those identified by the original two-class models (Fig. 3a, b) indicating that there was not a group of OTUs that clearly distinguished D from HD feet 2 + weeks before disease onset. There was also no significant difference in diversity between HD and D feet 2 + weeks before disease onset (Fig. 4 and Table S1), however, the Inverse Simpson index was significantly higher in H feet than HD and D feet. We conclude that in the period 2 + weeks before disease onset, IDS communities from sheep that developed footrot were different to those from sheep that remained healthy, but communities from HD and D feet were similar.

Within the seven differentiating OTUs, there were two members of the genus *Porphyromonas* and one from the genus *Prevotella* (Fig. 3c) both associated with disease in humans and sheep^21,22,24,34,35^. OTU_23 was a member of the genus *Pseudoclavibacter,* with occasional pathogenicity^36–38^, and we were unable to determine an accurate taxonomic identification for OTU_190, OTU_69 and OTU_364 using the Genbank database (Fig. S6).

### Diversity of the interdigital skin microbiota decreases prior to footrot

Linear mixed effects models of Inverse Simpson index showed that there was a reduction in OTU diversity on D feet one week before the onset of footrot, which resolved one week after footrot resolved (Fig. 4 and Table S2).

## Discussion

Our study provides the first evidence that dysbiosis of the IDS microbiota precedes development of footrot lesions. Dysbiosis occurs in two phases, initially sheep become predisposed to footrot, and then the microbiota changes again in the incubation period when feet become diseased. We discuss the IDS microbiota in healthy and predisposed sheep, and feet incubating footrot, and propose new hypotheses for the pathogenesis of footrot.

Sheep that developed footrot had a different IDS microbiota from sheep that remained healthy from the start of the study and before the incubation period for footrot; we hypothesise that this predisposed them to disease. It was not possible to determine whether this difference in community composition is permanent or temporary, only that it was present from the start of the study. The predisposed dysbiotic microbiota was less diverse than the healthy microbiota (Fig. 4) and varied from the healthy microbiota with the presence and abundance of seven OTUs out of over 2000 (Table 1 and Fig. 3c). This is a very small number of OTUs and with the model fitting approach used^33^, which minimises detection of false positive associations, it is highly likely these are true differences in the microbiota. Among the seven OTUs were *Porphyromonas* and *Prevotella* species. *Prevotella* and *Porphyromonas* are present in IDS of feet with footrot^21,22^ but their role in disease initiation was unknown. Both *Porphyromonas* and *Prevotella* subvert the host immune system^24,26,39,40^, and we hypothesise that they facilitate dysbiosis and a pro-inflammatory state which damages the integrity of the IDS and enables *D. nodosus* to invade the epidermis. This predisposing dysbiosis was present two or more weeks before footrot developed, longer than the 8–10 day incubation period of footrot^12,13^ indicating that it is likely to be causal, initiating footrot, rather than on the causal pathway. Further study of the functional activity of both *Porphyromonas* and *Prevotella* species prior to and during footrot episodes is now warranted.

In sheep that developed footrot there was a further reduction in bacterial diversity in D feet one week before clinical signs developed (Fig. 4). This is the incubation period when the load of *D. nodosus* increases^18^, it lasts 8–10 days^12,13^. The triggering event for the progression of disease on D feet compared to HD feet is not known. It is unlikely to relate to the environment or host susceptibility given that these factors will influence all feet of the same sheep equally. One possibility is that all feet of diseased sheep are susceptible to disease due to the composition of their IDS microbiota, and development of disease occurs if feet are contaminated with sufficient load of *D. nodosus*. Alternatively, strain-level or functional differences in the IDS microbiota that were not evident in 16S rRNA gene sequencing data could influence the initiation of footrot. Further study of the IDS microbiota during the 2 weeks prior to footrot using whole genome metagenomics and metatranscriptomics would help to address this question.

The IDS microbiota was temporally dynamic, with communities more similar in time across sheep than over time within sheep (Fig. 2). By accounting for confounding effects of the change in the IDS microbiota over time on diversity and community composition, we identified relationships between the microbiota and disease. We hypothesise that change in environmental conditions over time led to a change in OTUs on the IDS; throughout the twenty week study the soil moisture changed from wet to dry and the temperature gradually increased^19^. It is possible that environmental conditions facilitate dysbiosis by favouring colonisation with *Porphyromonas* and *Prevotella* spp; this would agree with existing knowledge that footrot is closely linked to the environmental conditions, in particular soil moisture^15,19,41–43^. It is also possible that different microbial communities respond differently to the same environmental stimuli; the interaction between the IDS microbiota and the environment would be a useful area of future study.

The current approach to management of footrot is to treat diseased sheep, our results suggest that managing the IDS microbiota could provide an alternative approach. This could be management interventions that favour development of a protective microbiota, or through targeting organisms such as *Porphyromonas* that contribute to disease pathogenesis. Vaccines against members of the *Porphyromonas* genus are being trialled for use in periodontitis^44^ and therefore a similar approach could be investigated for preventing footrot. A key step would be to identify whether establishment of the IDS microbiota at birth influences long term disease susceptibility as is reported for the gut microbiota^45^, or whether events such as changing environmental conditions trigger changes in the IDS microbiota. If the former were true, selection for resistant sheep based on the IDS microbiota might be possible. If only the latter were true, management of the environment might be useful. Clearly considerable work is required to develop these ideas but the novel findings in the current paper provide a new avenue to consider alternative approaches to present treatments.

## Conclusions

We conclude that there are two stages of dysbiosis of the interdigital skin: the first predisposes sheep to colonisation with *D. nodosus* and so initiates footrot, with remarkably only seven OTUs predicting susceptible sheep. The second arises in feet during incubation through to disease. These highly novel results further our understanding of the role of bacteria in the pathogenesis of footrot in sheep.

## Methods

### Study sample and data collection

Ethical approval for the studies was obtained from the University of Warwick’s local ethical committee; the Animal Welfare & Ethical Review Body (AWERB.33/13-14). All experiments were performed in accordance with relevant guidelines and regulations and the study is reported in accordance with ARRIVE guidelines.

Collection of samples and metadata for this study are described in^19^. Briefly, the data collection ran for 20 weeks from February to July 2015. In week 1 a group of 120 ten-month old Suffolk × Wiltshire Horn sheep from one flock were examined for footrot phenotype by scoring lesions of interdigital dermatitis (ID) and severe footrot (SFR) on a scale of 0 to 4 where 0 indicates no lesion and increasing scores indicate increasing severity^32^. Forty healthy sheep defined as ID score ≤ 1, SFR score = 0, were selected as the study sample. The interdigital skin (IDS) of each foot of the 40 sheep was swab sampled and then the sheep were immediately moved to a pasture that had not been stocked with any livestock for ≥ 10 days to ensure that it was free from *D. nodosus*^10^. IDS swab samples and footrot phenotype scores were collected from the 40 sheep each week from weeks 2 to 20. In addition, data on daily rainfall and temperature, and weekly soil moisture and temperature were collected^19^.

### Selection of a subset of sheep for sequencing analysis

Ten sheep, five that remained healthy and five that developed footrot at least once were selected for the current study based on observed foot scores (Fig. S1).There were 28/40 sheep that had at least one case of footrot; of these five were selected to provide a range of severities and durations of disease. Healthy sheep were defined as those with no occurrences of footrot; of these, the five with the highest number of occurrences of ID score = 0 were selected. Since unlike conventional statistical approaches, exact sample size calculations are problematic for machine learning models^46,47^, we based our sample size estimate on similar recent research^21^. In total we selected 620 samples from 10 sheep over 20 time points; McPherson et al.^21^ used 94 samples from 8 sheep over 10 time points and successfully identified significant differences in OTU abundance between healthy and footrot affected feet.

### Library preparation for 16S rRNA gene sequencing

DNA was extracted from IDS swabs using the method described by Purdy 2005^19,48^. The V1-V3 variable region of the bacterial 16S rRNA gene was amplified in a first stage PCR using the 27F-YM and 534R primers^49,50^ with overhanging adaptors for Illumina MiSeq sample preparation (Table S3). The primer sequences were based on those described for the Nextera XT DNA library preparation kit (Illumina, San Diego, CA) but the forward primer was adapted to 27F-YM^51^ to correct for mismatches with the *D. nodosus* 16S sequence^52^ (Table S3). Successful amplification of the *D. nodosus* VCS1703A strain 16S rRNA gene was confirmed. Prior to library preparation we also confirmed detection of *D. nodosus* with these primers by sequencing six IDS samples known to be positive for *D. nodosus* at high (n = 2), medium (n = 2) and low load (n = 2).

Following PCR amplification, amplicons were purified using Agencourt AMPure XP magnetic beads (Beckman-Coulter, Brea, CA), normalised using the SequelPrep Normalisation Plate kit (Applied Biosystems, Warrington, UK), and pooled into two libraries for sequencing using 300 bp paired-end Illumina MiSeq sequencing at the University of Warwick Genomics Facility.

### Extraction and PCR controls

An extraction control of sterile molecular biology grade water was used in all sets of DNA extractions. All PCR plates also included DNA from a model community as a positive control (*Staphylococcus aureus*, *Escherichia coli*, *Streptococcus agalactiae*, *Streptococcus dysgalactiae* and *Streptococcus uberis*) and a PCR negative control of sterile molecular biology grade water. All controls were sequenced alongside the samples.

### Processing of 16S rRNA gene sequencing data

Sequence data were processed using USEARCH version 8.1^53,54^. All samples had reverse reads truncated by 30 bp as per advice from Illumina at the time. Forward and truncated reverse reads were then merged, allowing for ≤ 2 mismatches. Samples were quality filtered using a maximum error rate of 0.5% and minimum sequence length of 467 bp. Data from the negative control samples were used to remove contaminant sequences (data filtered at 96% match), and the resulting filtered sequences were clustered into OTUs in USEARCH v8.1 at 97% similarity. An initial taxonomy was then assigned to each OTU in QIIME^55^ using the Greengenes database version 13.8^56^. For OTUs identified in the data analysis as predicting disease status, taxonomic assignment was confirmed using nucleotide BLAST against the rRNA Bacteria and Archaea database with uncultured sequences excluded.

### Statistical analysis of microbial communities

All analyses were conducted in the R statistical environment version 4.0.2^57^.

### Determination of beta diversity distance measures

Data were converted to relative abundance^58,59^ and pairwise dissimilarity between communities was calculated using four distance metrics (Root Jensen Shannon Distance (rJSD), Bray Curtis, and unweighted and weighted Unifrac) with the Phyloseq package in R^60^. Similarity between communities was visualised using principal coordinate analysis (PCoA).

Methods for determination of clustering based on beta diversity distance measures followed the principles described by Koren et al.^61^ and were adapted from the robust clustering algorithm developed by García-Jiménez and Wilkinson^62^. Clustering was performed using two approaches: the Partition Around Medoids (PAM) algorithm and hierarchical clustering. For each approach results were compared for the four distance metrics described above. Two types of clustering score (average Silhouette width (SI) and Prediction Strength (PS)) followed by an additional bootstrapping process (evaluated with the Jaccard similarity score) were used to determine whether there were distinct microbiome states (thresholds as described by Koren et al.^61^), and to identify the optimum number of clusters (Fig. S7).

### Use of machine learning to identify OTUs predictive of subsequent disease status

All models were implemented using the caret package^63^. Prior to implementation of each model, unsupervised filtering was used to remove rare OTUs (those with fewer than 10 reads or present in fewer than 5% of samples). All predictors were standardised (mean subtracted and then divided by standard deviation) and those with near-zero variance (< 10% distinct values out of number of total samples and ratio of the frequency of the most common value to the frequency of the second most common value > 95/5) were removed.

A random forest regression with week of study as a continuous outcome was used to identify and remove 140 OTUs highly correlated with time. Only data from healthy sheep were included in this analysis (218 observations) and after filtering OTU data as described above, 2319 OTUs were offered to the model. The model was fit to the data and hyperparameters optimised using leave-one-out cross-validation (data from one sheep left out each time). Visual assessment of the variable importance plot for the model was used to identify the OTUs to be removed (Fig. S8).

**Figure 5:**
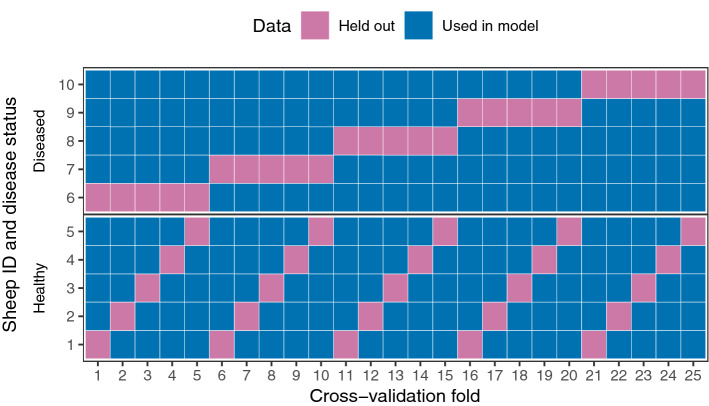
Schematic of cross-validation for machine learning models. The figure illustrates how data from each sheep were partitioned into modelling and holdout sets based on leaving out each of the 25 possible combinations of one healthy and one diseased sheep.

Two machine learning algorithms were used for classification of disease status: random forest (RF) and stochastic gradient boosting (GBM). The outcome variable of interest was whether a sheep later developed footrot or never developed footrot. After filtering out rare OTUs and those with near zero variance, there were 2184 OTUs offered to each algorithm. A common approach to implementation of each algorithm was used as follows. First, each model was fit to the data and hyperparameters optimised using cross-validation with 25 folds created by leaving out each possible combination of one healthy and one diseased sheep (Fig. 5). For each algorithm, recursive feature elimination (RFE)^64^ was used to identify the smallest subset of features that was required for classification without compromising accuracy (Fig. S9). Subset sizes tested were 10, 20, 30, 40, 50, 100, 500, 1000, 1500, 2000 and 2184 features. An accuracy value that was within 5% of the maximum value was tolerated. The *N* most important features where *N* was the optimum subset size were identified for each algorithm based on feature rankings across all resampling iterations, and a reduced dataset was then created containing only those OTUs identified by both RF and GBM algorithms. This comprised seven OTUs. Classification was repeated with both algorithms using only seven OTUs to confirm that using the reduced dataset did not decrease model performance.

#### Repeating machine learning analyses using a three-class model

To determine whether a three-class model better fit the data, a random forest algorithm was used to classify samples as belonging to H, D or HD feet using methods described above. The same 2184 OTUs were offered to the algorithm as for the two-class model described above and the model was fit using the same cross-validation procedure (Fig. 5). Model performance was assessed using a confusion matrix to determine the proportion of each foot status group (H, D, HD) correctly identified in cross-validation. The top ten most important features from the model were compared to the most important features for the two-class model.

#### Repeating machine learning analyses with load of Dichelobacter nodosus included

There were 20 samples with high loads of *D. nodosus* (between 10^5^ and 10^7^ *rpoD* gene copies μl^-1^) where the *D. nodosus* OTU was not detected within our data (Fig. S10). We confirmed that sequences belonging to *D. nodosus* were not wrongly assigned or filtered out during quality control, and so we conclude that there were mismatch issues with the V1–V3 primers. Given our primers successfully amplified the VCS1703A strain, we propose that strain level variability may occur at this region meaning that some strains are amplified more than others.

To test whether failure to detect the *D. nodosus* OTU impacted on model accuracy for predicting disease status, we repeated the machine learning analysis described above with bacterial load of *D. nodosus* included as an additional predictor variable. For the 194 samples where qPCR data was not available, load of *D. nodosus* was imputed using the Expectation–Maximisation with Bootstrapping algorithm^65^ in the Amelia II package^66^. Our results confirmed that inclusion of bacterial load of *D. nodosus* did not change model performance.

### Determination of changes in alpha diversity with disease status

The Inverse Simpson index was estimated for all samples using the function provided within the Phyloseq package^60^. A linear mixed-effects model implemented in the lme4 package^67^ was used to investigate associations between the Inverse Simpson index and disease status of feet. Inverse Simpson index was right skewed and was therefore square root transformed prior to analysis (Fig. S11). A categorical variable for disease status was tested as a fixed effect with the following seven categories: H feet, HD feet, D feet 2 + weeks before onset of footrot, D feet 1 week before onset of footrot, D feet during a footrot episode, D feet 1 week after a footrot episode ended, and D feet 2 + weeks after a footrot episode ended. Sheep was included as a random effect to account for clustering of feet within sheep and a third order polynomial term for day of study was included as a fixed effect to account for underlying changes in diversity over time. Fitted versus residuals plots were visually assessed to check for non-linearity, unequal variances and outliers (Fig. S12), and posterior predictive simulation^67,68^ was used to check model fit.

## Supplementary Information

Supplementary Information.

## Data Availability

The datasets generated during the current study are available in the NCBI SRA repository, https://www.ncbi.nlm.nih.gov/sra/PRJNA772107.
