# Supplementary material for: Differences in composition of interdigital skin microbiota predict sheep and feet that develop footrot
Source: Sci Rep. 2022 May 27;12:8931. doi: 10.1038/s41598-022-12772-7 (PMC9142565; doi:10.1038/s41598-022-12772-7)
Supplement: Supplementary file 1 — Supplementary Information. [file 41598_2022_12772_MOESM1_ESM.pdf]

Supplementary information: Differences in composition of interdigital skin microbiota predict sheep and feet that develop footrot

Rachel Clifton, Emma M. Monaghan, Martin J. Green, Kevin J. Purdy and Laura E. Green

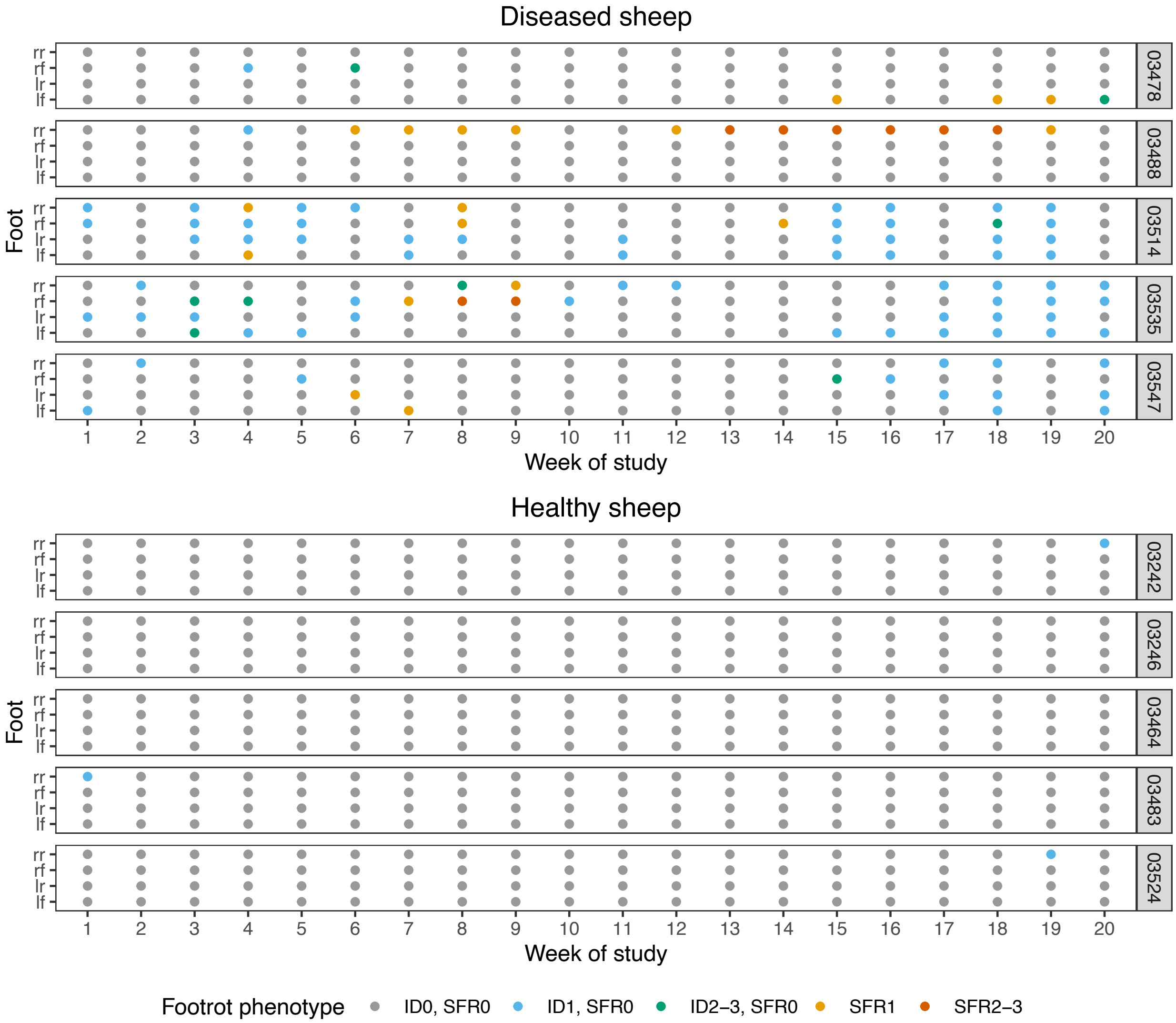

Figure S1 Footrot phenotype by foot and week for five diseased sheep and five healthy sheep. ID = interdigital dermatitis, SFR = severe footrot, lf = left fore, lr = left rear, rf = right fore, rr = right rear, 03*** = sheep ID.

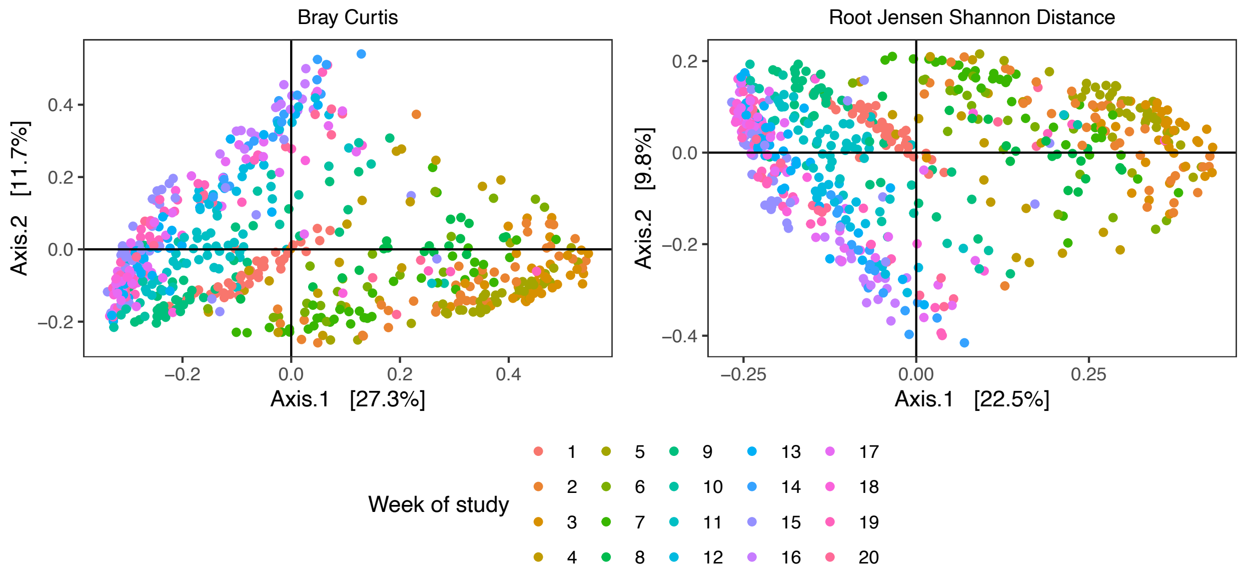

Figure S2 Temporal changes in interdigital skin microbial community composition. Principal coordinate analysis (PCoA) plot of Bray Curtis and root Jensen Shannon Distance dissimilarities between microbial communities at the operational taxonomic unit (OTU) level. Each dot represents a sample coloured by week of study. Number of samples (n) = 603.

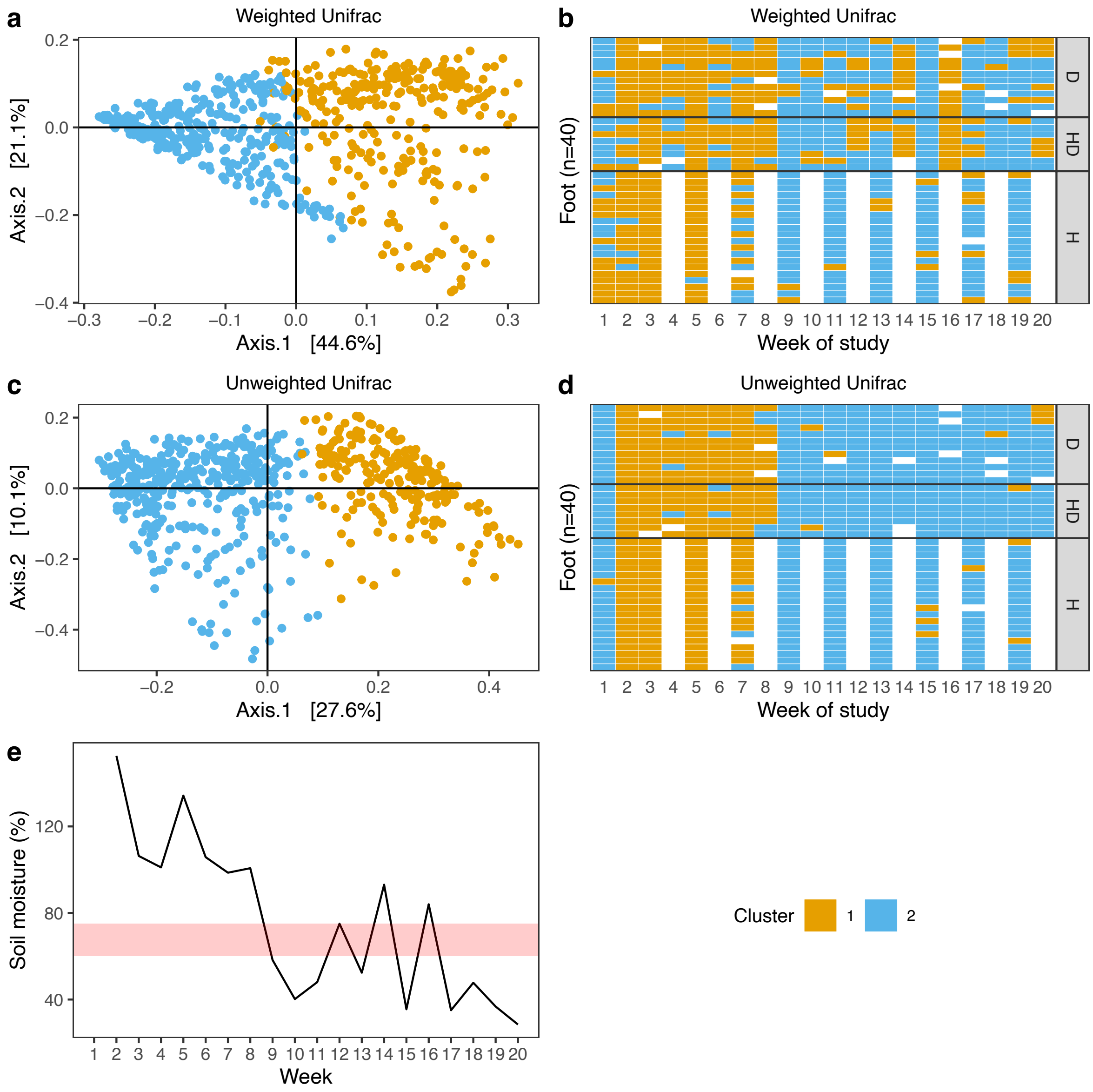

Figure S3 Presence of two microbiome states temporally associated with changes in soil moisture. a, c Principal coordinate analysis (PCoA) plot of weighted and unweighted Unifrac dissimilarities between samples at the operational taxonomic unit (OTU) level. Each dot represents a sample. Color indicates microbiome state as determined by clustering using a partition around medoids (PAM) algorithm. **b, d** Transition between microbiome states over time for 40 feet using weighted and unweighted Unifrac distance measures. Each row represents an individual foot and colour indicates microbiome state. White space indicates a missing sample or a sample that was not selected for analysis. **e,** Mean moisture content of surface soil by week of study (three samples per week), showing a sharp decrease in moisture content between weeks 8 and 10 of the study. Red band shows potential threshold for transition between microbiome states with weighted Unifrac measure. For **a, b, c** and **d** number of samples (n) = 603.

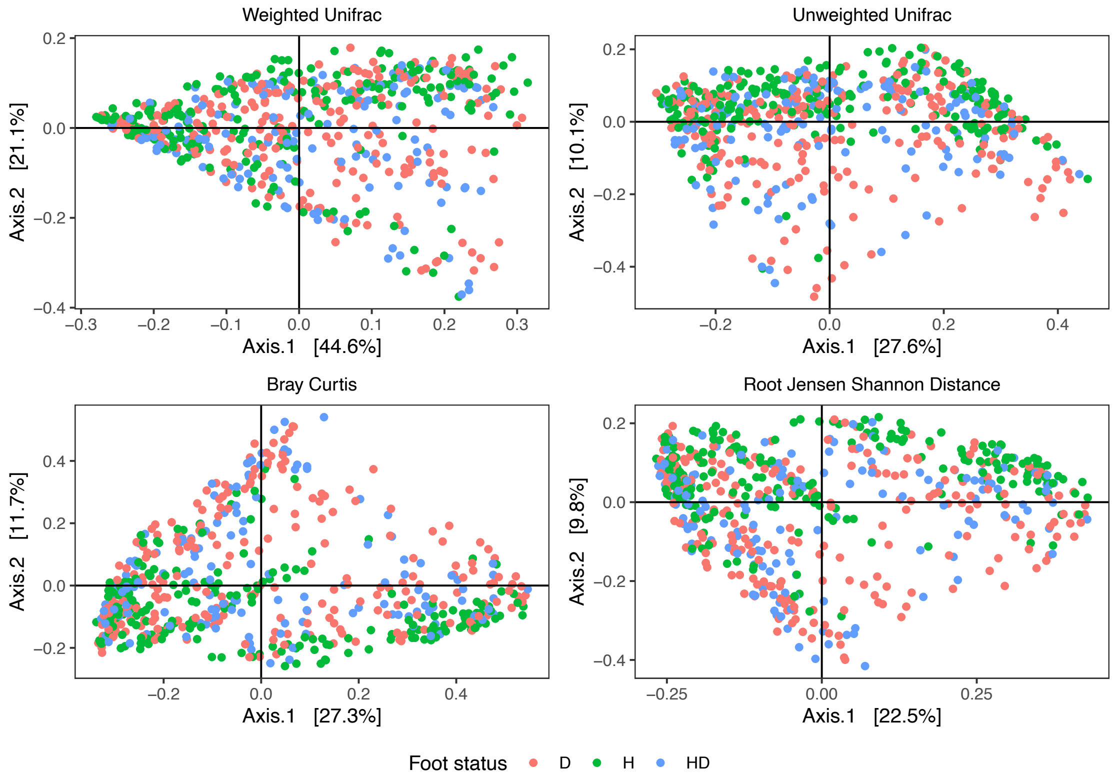

Figure S4 No evidence for clustering of microbial communities by foot status. Principal coordinate analysis (PCoA) plot of weighted and unweighted Unifrac, Bray Curtis and root Jensen Shannon Distance dissimilarities between microbial communities at the operational taxonomic unit (OTU) level. Each dot represents a sample coloured by foot status: H = healthy foot of healthy sheep; HD = healthy foot of diseased sheep; D = diseased foot. Number of samples (n) = 603.

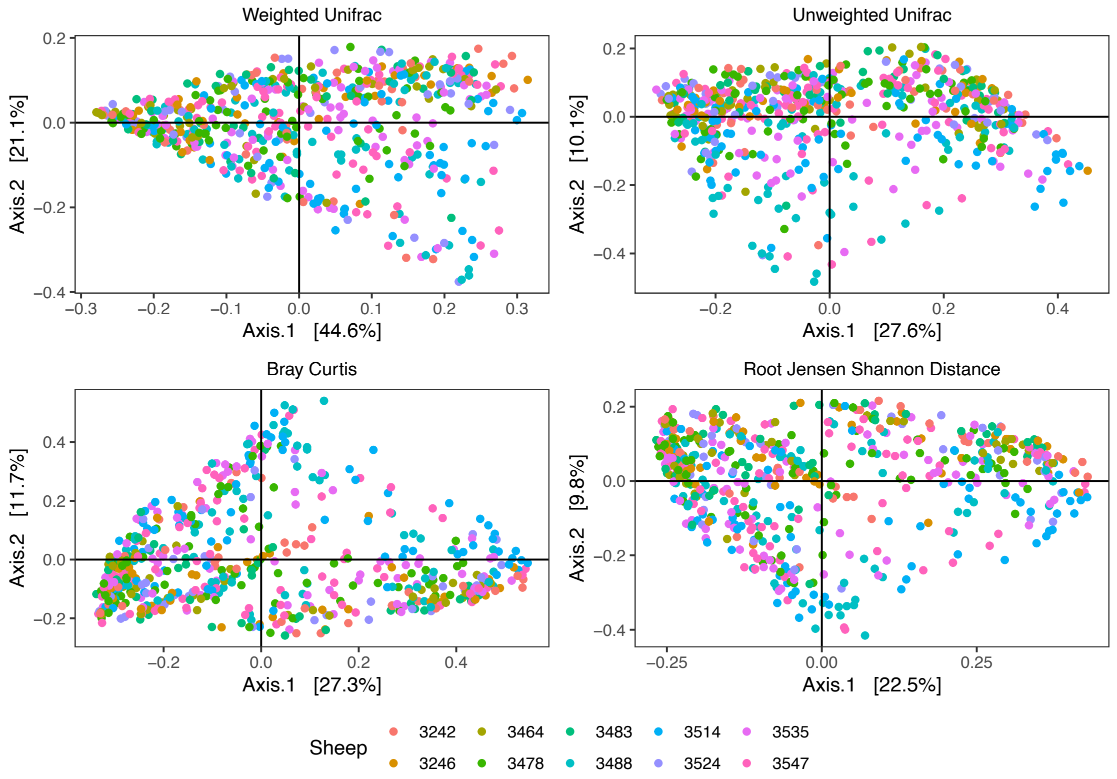

Figure S5 No evidence of clustering of microbial communities by sheep. Principal coordinate analysis (PCoA) plot of weighted and unweighted Unifrac, Bray Curtis and root Jensen Shannon Distance dissimilarities between microbial communities at the operational taxonomic unit (OTU) level. Each dot represents a sample coloured by sheep ID. Number of samples (n) = 603.

Table S1 Results of linear mixed effects regression model of square root transformed Inverse Simpson Index with H feet as reference category for foot status. Number of samples (n) = 603.

Variable	Coefficient	Lower CI[a]	Upper CI[a]	P value
Foot status				
H	Ref			
HD	-36.957	-46.75	-27.164	4.72e-13
D before 2+	15.159	6.278	24.04	0.000873
D before 1	5.972	-2.905	14.849	0.188
D during	-2.616	-3.779	-1.453	0.000291
D after 1	-2.597	-4.095	-1.098	0.00133
D after 2+	-5.304	-8.01	-2.599	0.000146
Polynomial term for days of study				
Days ^1	-5.427	-7.242	-3.612	5.41e-08
Days ^2	-5.484	-7.883	-3.085	1.14e-05
Days ^3	-2.663	-3.957	-1.37	0.000382

Variance of sheep level random effects = 0.301

Residual variance = 20

[a] 95% Confidence interval (Wald)

OTU_364

Description	Scientific Name	Max Score	Total Score	Query Cover	E value	Per. Ident	Acc. Len	Accession
Helcococcus sueciensis strain type strain: CCUG 47334 16S ribosomal RNA, partial seque...	Helcococcus sueciensis	604	604	99%	1e-172	88.10%	1512	NR_025606.1
Helcococcus kunzii strain 22 16S ribosomal RNA, partial sequence	Helcococcus kunzii	597	597	100%	2e-170	87.55%	1465	NR_029237.1
Helcococcus ovis strain s840-96-2 16S ribosomal RNA, partial sequence	Helcococcus ovis	597	597	97%	2e-170	88.24%	1423	NR_027228.1
Helcococcus seattlensis strain F5780 16S ribosomal RNA, partial sequence	Helcococcus seattlensis	580	580	100%	2e-165	87.05%	1529	NR_118641.1
Tissierella carlieri strain LBN 295 16S ribosomal RNA, partial sequence	Tissierella carlieri	562	562	99%	7e-160	86.46%	1415	NR_133054.1
Anaerococcus vaginalis strain GIFU 12669 16S ribosomal RNA, partial sequence	Anaerococcus vaginalis	558	558	100%	9e-159	85.91%	1375	NR_115508.1
Anaerococcus rubeinfantis strain mt16 16S ribosomal RNA, partial sequence	Anaerococcus rubeinfantis	556	556	100%	3e-158	86.37%	1490	NR_144730.1
Tissierella praeacuta strain ATCC 25539 16S ribosomal RNA, partial sequence	Tissierella praeacuta	553	553	100%	4e-157	85.99%	1475	NR_119111.1
Proteiniborus ethanoligenes strain GW 16S ribosomal RNA, partial sequence	Proteiniborus ethanoligenes	553	553	100%	4e-157	86.15%	1523	NR_044093.1
Tissierella praeacuta strain ATCC 25539 16S ribosomal RNA, partial sequence	Tissierella praeacuta	551	551	99%	2e-156	86.10%	1415	NR_117376.1

OTU_190

Description	Scientific Name	Max Score	Total Score	Query Cover	E value	Per. Ident	Acc. Len	Accession
Gottschalkia purinilytica strain DSM 1384 16S ribosomal RNA, partial sequence	Gottschalkia purinilytica	616	616	99%	6e-176	88.37%	1517	NR_117121.1
Gottschalkia purinilytica strain ATCC 33906 16S ribosomal RNA, partial sequence	Gottschalkia purinilytica	612	612	100%	7e-175	88.05%	1470	NR_118735.2
Andreesenia angusta strain MK-1 16S ribosomal RNA, partial sequence	Andreesenia angusta	604	604	100%	1e-172	87.93%	1464	NR_044642.2
Sporanaerobacter acetigenes strain Lup33 16S ribosomal RNA, partial sequence	Sporanaerobacter acetigenes	588	588	100%	1e-167	87.28%	1523	NR_025151.1
Sporanaerobacter acetigenes strain DSM 13106 16S ribosomal RNA, partial sequence	Sporanaerobacter acetigenes	582	582	99%	6e-166	87.21%	1414	NR_117381.1
Gottschalkia acidurici strain 9a 16S ribosomal RNA, partial sequence	Gottschalkia acidurici	580	580	100%	2e-165	87.12%	1513	NR_074549.1
Gottschalkia acidurici strain DSM 604 16S ribosomal RNA, partial sequence	Gottschalkia acidurici	573	573	99%	3e-163	87.02%	1513	NR_117601.1
Proteiniborus ethanoligenes strain GW 16S ribosomal RNA, partial sequence	Proteiniborus ethanoligenes	569	569	100%	4e-162	86.65%	1523	NR_044093.1
Clostridiisalibacter paucivorans strain 37HS60 16S ribosomal RNA, partial sequence	Clostridiisalibacter paucivorans	566	566	98%	6e-161	86.99%	1434	NR_044043.1
Helcococcus ovis strain s840-96-2 16S ribosomal RNA, partial sequence	Helcococcus ovis	564	564	97%	2e-160	87.08%	1423	NR_027228.1

OTU_69

Description	Scientific Name	Max Score	Total Score	Query Cover	E value	Per. Ident	Acc. Len	Accession
Pedobacter nanyangensis strain Q-4 16S ribosomal RNA, partial sequence	Pedobacter nanyangensis	401	401	100%	2e-111	80.92%	1481	NR_137392.1
Acetobacteroides hydrogenigenes strain RL-C 16S ribosomal RNA, partial sequence	Acetobacteroides hydrogenigenes	390	390	96%	4e-108	81.15%	1440	NR_133950.1
Pricia antarctica strain ZS1-8 16S ribosomal RNA, partial sequence	Pricia antarctica	387	387	100%	5e-107	80.34%	1493	NR_108451.1
Sediminibacter furfurosus strain NBRC 101622 16S ribosomal RNA, partial sequence	Sediminibacter furfurosus	385	385	96%	2e-106	80.87%	1447	NR_114008.1
Sediminibacter furfurosus strain MAOS-86 16S ribosomal RNA, partial sequence	Sediminibacter furfurosus	385	385	96%	2e-106	80.87%	1444	NR_041452.1
Phaeocystidibacter luteus strain PG2S01 16S ribosomal RNA, partial sequence	Phaeocystidibacter luteus	381	381	100%	2e-105	80.19%	1480	NR_132329.1
Pelobium manganitolerans strain YS-25 16S ribosomal RNA, partial sequence	Pelobium manganitolerans	379	379	100%	8e-105	79.96%	1481	NR_153691.1
Negadavirga shengliensis strain SLG210-21 16S ribosomal RNA, partial sequence	Negadavirga shengliensis	379	379	96%	8e-105	80.59%	1446	NR_136439.1
Muricauda nanhaiensis strain SM1704 16S ribosomal RNA, partial sequence	Muricauda nanhaiensis	363	363	100%	8e-100	79.55%	1512	NR_171527.1
Hoppeia youngheungensis strain YIK12 16S ribosomal RNA, partial sequence	Hoppeia youngheungensis	359	359	97%	1e-98	79.77%	1489	NR_126298.1

Figure S6 Ten most similar sequences from nucleotide BLAST query for OTU_364, OTU_190 and OTU_69. Sequences were queried against the rRNA Bacteria and Archaea database with uncultured sequences excluded.

Table S2 Results of linear mixed effects regression model of square root transformed Inverse Simpson Index with D before 2+ feet as reference category for foot status. Number of samples (n) = 603.

Variable	Coefficient	Lower CI[a]	Upper CI[a]	P value
Foot status				
D before 2+	Ref			
D before 1	-2.708	-5.497	0.082	0.058
D during	-2.831	-4.842	-0.82	0.006
D after 1	-2.888	-5.427	-0.349	0.026
D after 2+	-0.067	-1.648	1.515	0.934
HD	-0.019	-1.453	1.415	0.979
H	2.597	1.098	4.095	0.001
Polynomial term for days of study				
Days ^1	-36.957	-46.75	-27.164	4.72e-13
Days ^2	15.159	6.278	24.04	0.001
Days ^3	5.972	-2.905	14.849	0.188

Variance of sheep level random effects = 0.301

Residual variance = 20

[a] 95% Confidence interval (Wald)

Table S3. Sequence for PCR primers used in 16S rRNA gene sequencing

Primer	Sequence (5’ – 3’)	PCR product size (bp)
27F-YM	AGAGTTTGAT**YM**TGGCTCAG	500
534R	ATTACCGCGGCTGCTGG	
**Primers with Illumina read sequence adaptors**		
27F-YM_rd1	**TCGTCGGCAGCGTCAGATGTGTATAAGAGACAG** AGAGTTTGATYMTGGCTCAG[a]	574
534R_rd2	**GTCTCGTGGGCTCGGAGATGTGTATAAGAGACAG**ATTACCGC GGCTGCTGG[a]	
**Primers with Illumina indexes**		
Index N7	**CAAGCAGAAGACGGCATACGAGAT**NNNNNNNN*GTCTCGTGG GCTCGG*[b]	643
Index S5	**AATGATACGGCGACCACCGAGATCTACAC**NNNNNNNN*TCGTC GGCAGCGTC*[b]	

[a] Illumina read sequence adapter is in bold and underlined.

[b] Indexing primers consist of a sequencing adapter (bold and underlined), a unique 8 base index (represented by N’s) and a sequence complementary to the read sequence adapters (in italics).

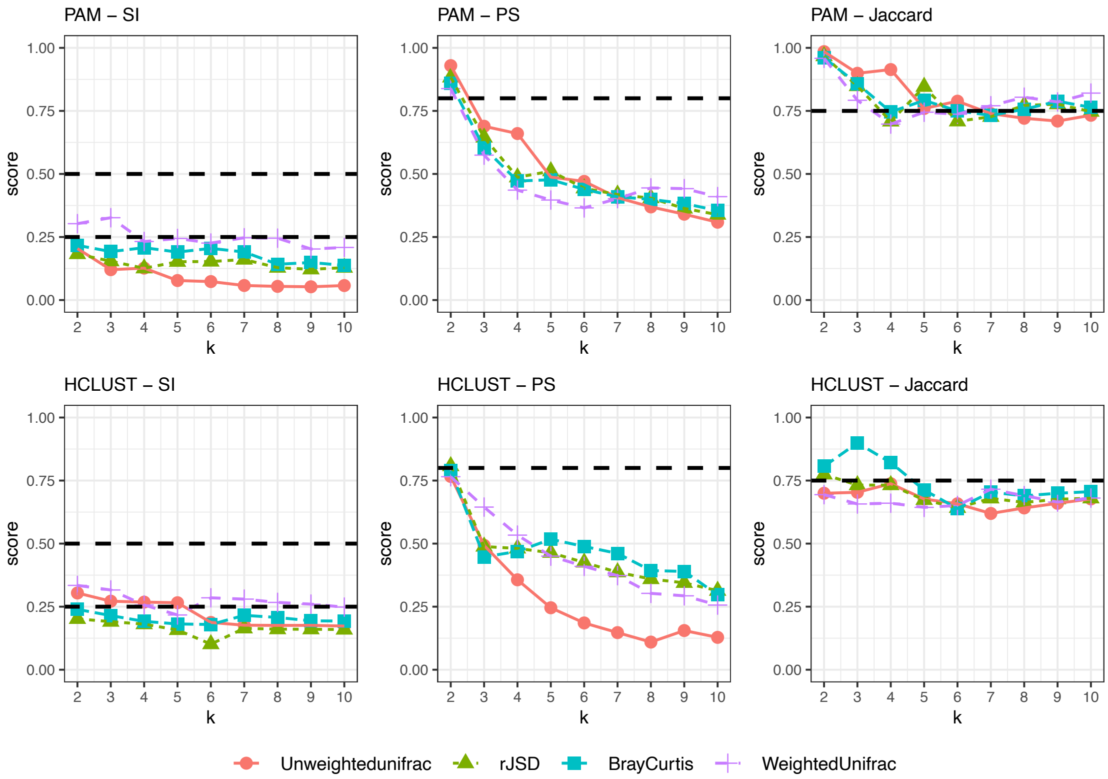

Figure S7 Evaluation of clustering with four distance metrics in 603 IDS swab samples. Top row: clustering assessed using partition around medoids (PAM) algorithm. Bottom row: clustering assessed using hierarchical clustering (HCLUST). SI = Average Silhouette width, PS = Prediction Strength and Jaccard = Jaccard similarity score for bootstrapping. Distance metrics: unweighted Unifrac, root Jensen Shannon Distance, Bray Curtis and weighted Unifrac.

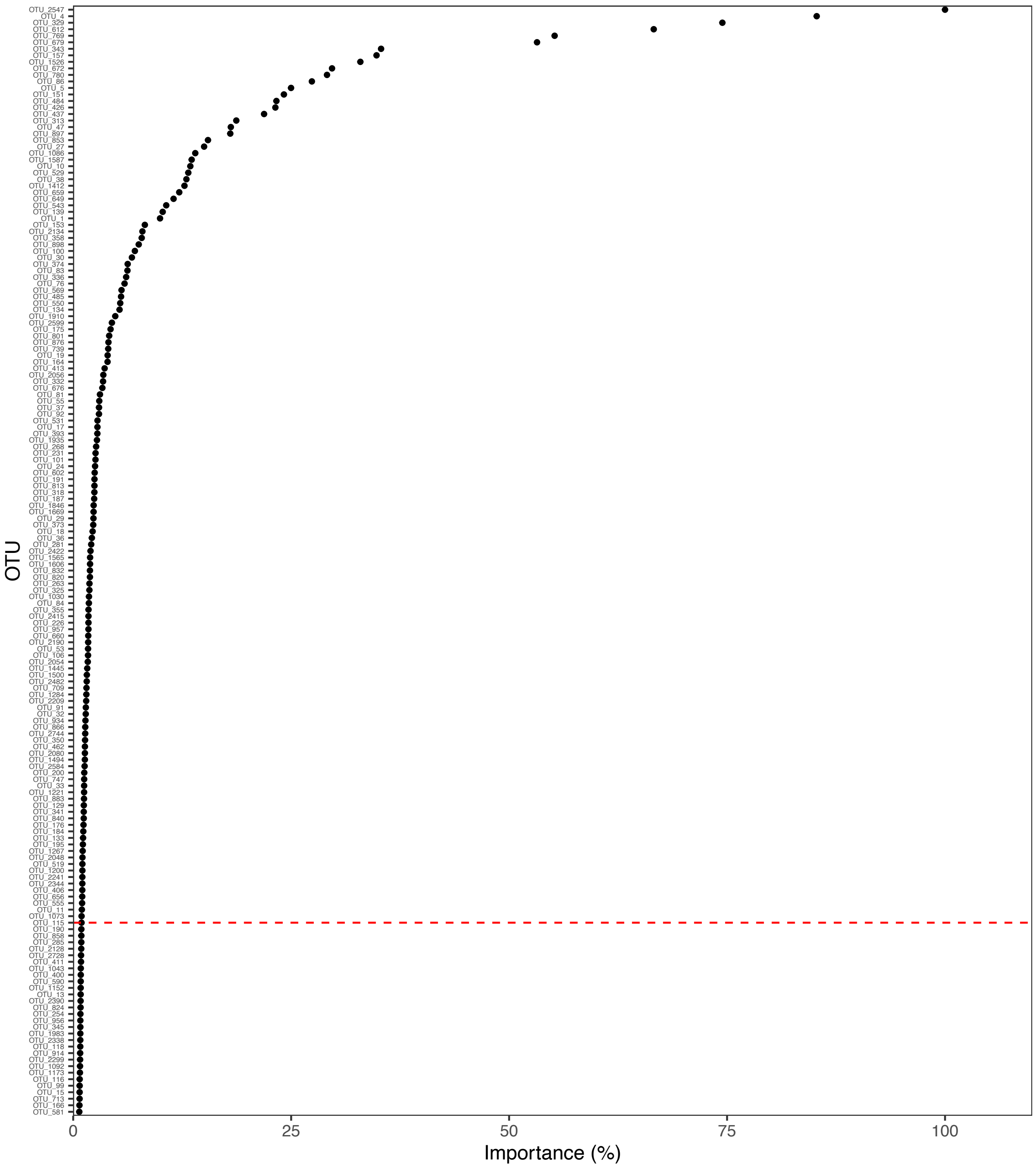

Fig S8 Importance of 170 most important OTUs in random forest regression model of week of study using data from healthy sheep. Importance is scaled as a percentage relative to the importance of the most important OTU. Dashed red line shows cut-off for top 140 OTUs removed prior to analysis of disease status. Number of samples (n) = 218.

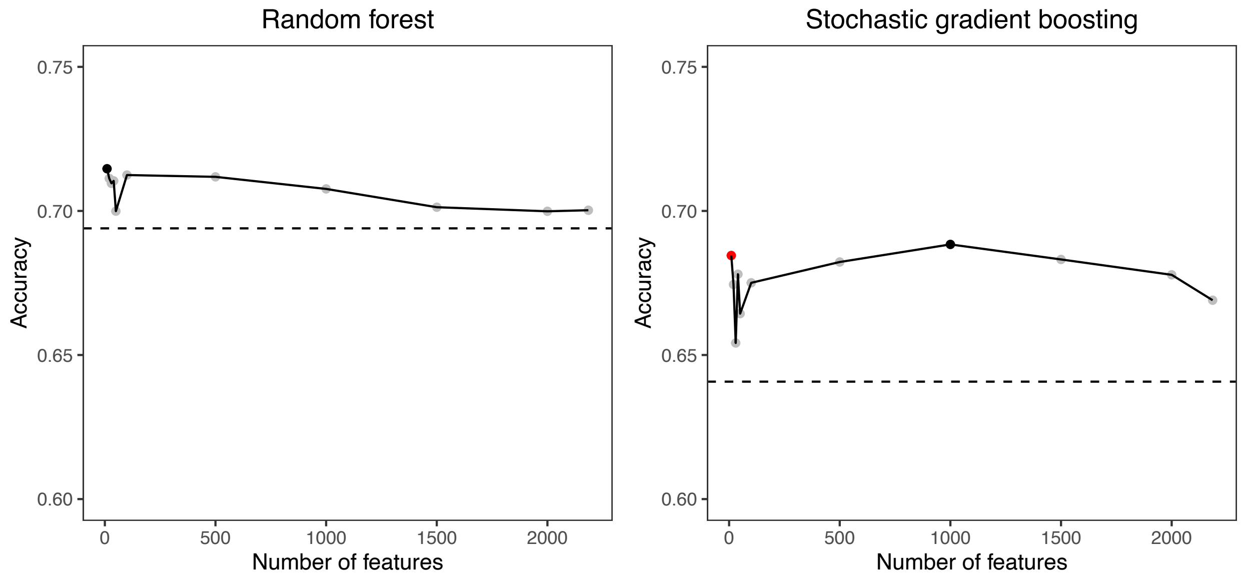

Figure S9 Accuracy of random forest (left panel) and stochastic gradient boosting (right panel) algorithms for predicting disease status of sheep by number of features offered to model. Black circle shows number of features resulting in maximum accuracy and red circle shows smallest number of features resulting in an accuracy value within 5% (dashed line) of the maximum value (if applicable). Subset sizes tested were 10, 20, 30, 40, 50, 100, 500, 1,000, 1,500 and 2,000 and 2,184 features. Number of samples (n) = 117.

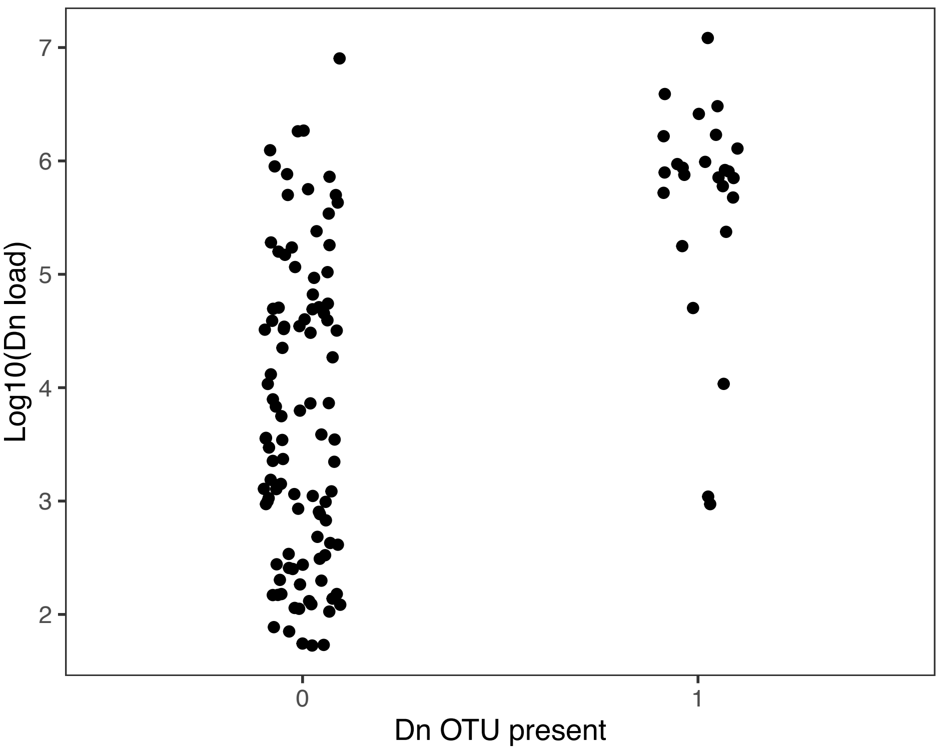

Figure S10 Load of *Dichelobacter nodosus* by presence of the *Dichelobacter nodosus* OTU. Load was measured using qPCR. Only samples with load > 0 are shown (n = 126).

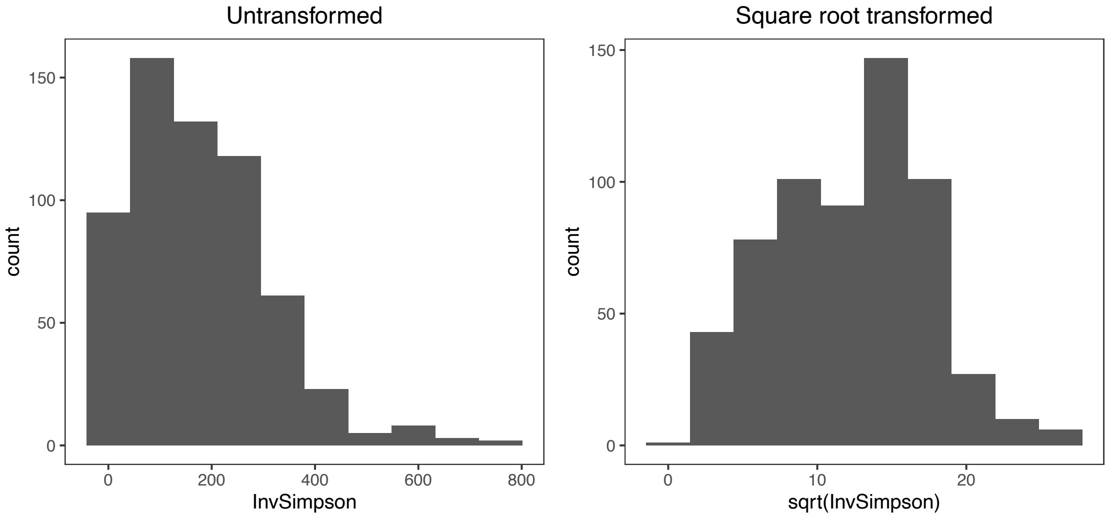

Figure S11 Distribution of untransformed and square root transformed Inverse Simpson Index data from 603 IDS samples.

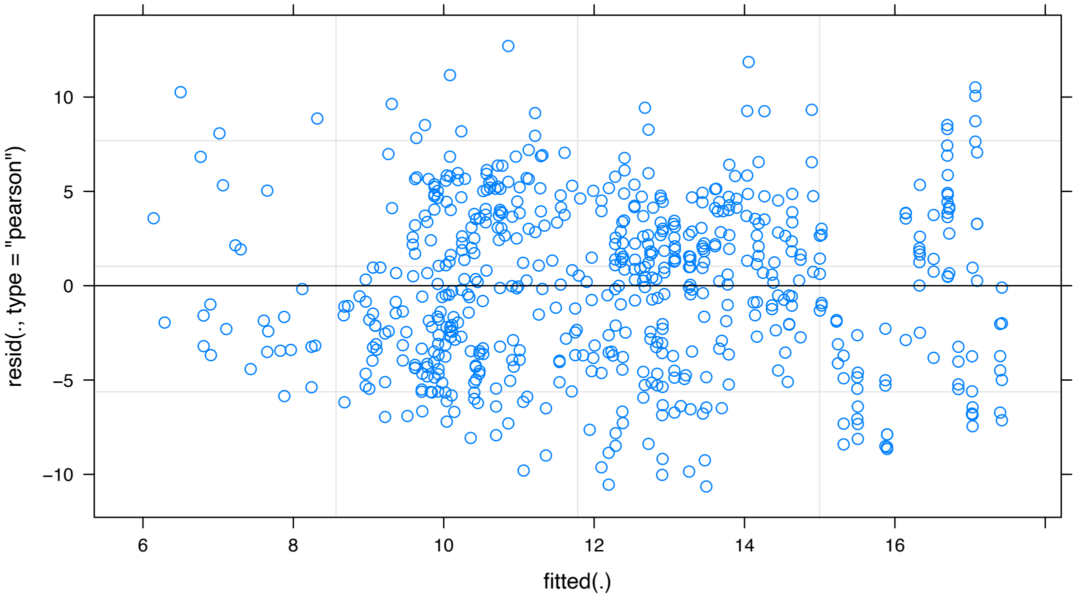

Figure S12 Residuals versus fits plots for a final linear mixed effects model for square root transformed Inverse Simpson Index. Number of samples (n) = 603.
